# Impact of a Real-Time Virtual Rounding Queue on Neonatal Intensive Care Unit Rounding Practices: Survey Study of Clinicians’ Perceptions

**DOI:** 10.2196/78547

**Published:** 2026-06-18

**Authors:** Elizabeth S Jarrett, Alexis Quade, Johannah M Scheurer, Michael B Pitt, John Sartori, Scott Lunos, Tamara Kasal, Rubina F Rizvi

**Affiliations:** 1 Department of Pediatrics, University of Minnesota, 2450 Riverside Ave, Minneapolis, MN, 55454, United States, 612-365-6777; 2 Department of Medicine, Division of Hospital Medicine, University of Minnesota, , Minneapolis, MN, United States; 3 Department of Electrical and Computer Engineering, University of Minnesota, Minneapolis, MN, United States; 4 Biostatistical Design and Analysis Center, Clinical and Translational Science Institute, University of Minnesota, Minneapolis, MN, United States; 5 Fairview Health Services, Minneapolis, United States; 6 Center for Learning Health Systems, Division of Computational Health Sciences, Department of Surgery, University of Minnesota, Minneapolis, IL, United States

**Keywords:** electronic health records, neonatal intensive care unit, standard of care, surveys and questionnaires, software

## Abstract

**Background:**

While family-centered rounds are considered the standard of care, coordinating the presence of all stakeholders without knowing when the medical team will arrive can make their implementation challenging. To address this barrier, we created novel software, Q-rounds, that is integrated into the electronic health record and creates a real-time rounding queue that updates nurses and family members on medical team rounding time and allows families to join in person or remotely via phone. A previous observational study showed that the implementation of Q-rounds in a neonatal intensive care unit (NICU) led to increased nurse and family presence.

**Objective:**

This study aimed to understand clinicians’ perceptions of the use of Q-rounds on NICU rounding practices, such as the impact on rounding efficiency and clinician satisfaction, as well as facilitators and barriers in its implementation.

**Methods:**

Q-rounds was implemented in the NICU at M Health Fairview Masonic Children’s Hospital in May 2023. In this survey study, surveys were distributed to physicians, advanced practice providers, and nurses 6 weeks before implementation and 6 weeks afterward to collect data on the impact of Q-rounds on family-centered rounds. Data were analyzed both quantitatively and qualitatively.

**Results:**

There were 118 respondents in the preimplementation phase and 110 in the postimplementation phase. After implementation of Q-rounds, clinicians perceived an increase in nurse presence (“most” or “almost all of the time” n/N= 48/105, 46% to n/N=24/36, 75%; *P*<.001) and family presence (at least “sometimes” n/N=45/106, 46% to n/N=35/36, 97%; *P*<.001) on rounds. Respondents perceived rounds as more efficient (n/N=56/105, 53% to n/N=27/36, 75%; *P*=.006), and more respondents indicated being satisfied with rounds (n/N=59/105, 46% to n/N=62/84, 74%; *P*=.003). There was no perceived difference in rounding duration. These findings were supported by thematic analysis of open-ended responses.

**Conclusions:**

A novel virtual rounding queue software that notifies families and nurses of when to expect the rounding team was associated with increased clinician perceptions of efficiency, participation in rounds by nurses and families, and satisfaction with rounds.

## Introduction

### Study Background

The practice of multidisciplinary family-centered rounds (FCRs) is considered standard practice by the American Academy of Pediatrics [1,2]. FCR entails the care team, including physicians, trainees, bedside nurses, and other staff (eg, dieticians, respiratory therapists, and interpreters where needed), along with the family or caregivers, working together to set the daily plan of care. Ample evidence supports the benefits of FCR, with previous studies showing improvements in health care team communication [1,3-6], increased participation and decision-making of patients and families [7,8], increased family confidence in the health care team [7], improved patient safety [1,5], improved perception of teaching [5], and improved rounding efficiency [1]. Furthermore, FCR in the pediatric setting has been found to increase “humanistic outcomes” in trainees, including empathy, partnership, and respect [6].

Despite these known benefits to FCR, there are many barriers to implementation. Practically speaking, no matter how impactful FCR is, the improved outcomes can only be realized if the nurse and family are able to be present for rounds. In a scoping review on FCR’s implementation, the study by Knighton and Bass [2] reports that the “scheduling challenges for nurses, families, and others that want to attend rounds but may not have the flexibility needed because of important competing demands” is a significant barrier to implementation. Without knowing when to expect the team to arrive for a given patient, nurses are unable to plan for attendance or may be asked to drop what they are doing to join. Families often must choose between work or other obligations or sitting at the bedside for hours waiting their turn for rounds [9]. Additional barriers exist for families who communicate in languages other than English [2], which often has its own scheduling and logistics challenges [2,9]. Additionally, some medical teams are hesitant to implement FCR because of concerns regarding FCR taking more time than traditional rounds and potentially negatively affecting trainee teaching.

Other institutions have implemented static interventions to address scheduling barriers to FCR with some success, although they have their limitations. For instance, the study by Bekmezian et al [3] described implementing a rounding coordinator to complete the schedule and communication regarding rounding appointments and reported that this approach increased nurse attendance on rounds and resulted in positive feedback from patients and families. Other groups have implemented a rounding checklist to increase family participation [10] or have found providing a fixed rounding schedule before rounds began to be beneficial for increasing nurse and family presence [11,12]. Only 1 study addressed interpreter attendance [3], while many studies do not include patients or families who speak a language other than English. While it is promising that the barriers to FCR could be overcome, many of these strategies are prohibitively labor-intensive. Additionally, the use of a fixed rounding schedule also does not address the unpredictability of rounds, with often changing schedules as acute medical issues come up that need to be addressed.

### Preliminary Studies

**Figure 1.**
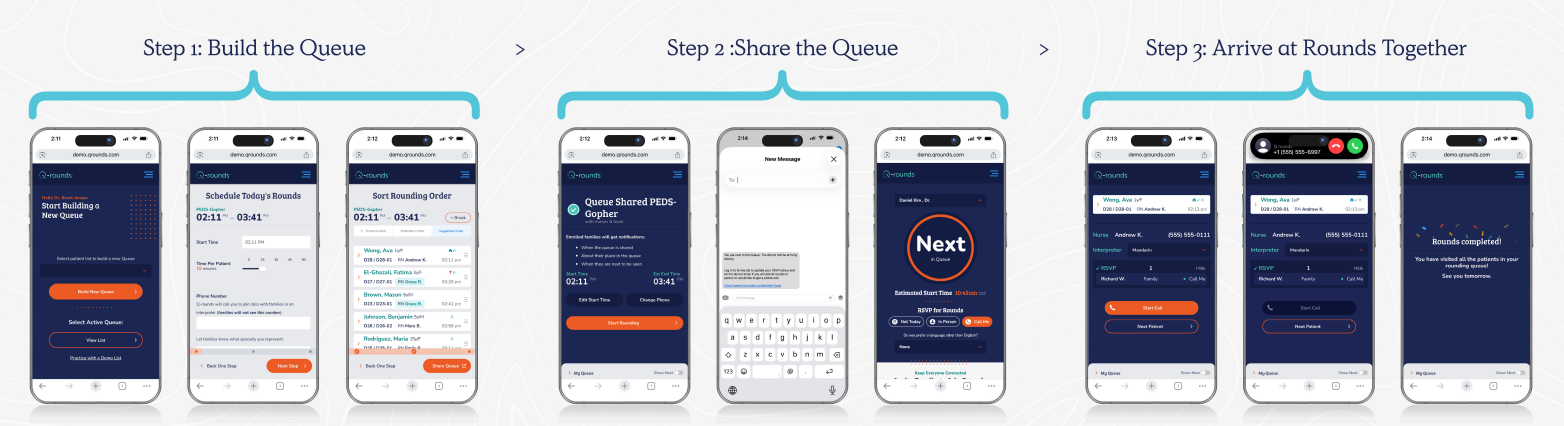
Overview of the workflow of the Q-rounds software.

This software was first implemented in our medical center’s neonatal intensive care unit (NICU). In an observational study comparing preimplementation and postimplementation of Q-rounds software (Figure 1), nurse and family attendance significantly increased without affecting the duration of rounds, with parent attendance increasing by over 100% [13]. However, this previous observational study did not address feedback from important members of the medical team, such as nurses, and the impact of the software on their experience with FCR. As the software was implemented, outcomes (eg, attendance of rounds by the key personnel, round planning, and efficiency) were incorporated in the deployment of the tool to capture the intervention’s impact and sustainability [14].

The physician or advanced practice provider starts by building a rounding queue based on anticipated clinical needs. They first estimate the rounding time for each patient. Next, the health care professional shares the rounding queue with the electronic health record (EHR) for other team members to view. The program calls nurses and texts the patients’ families to provide their patient’s spots in the rounding queue. When the rounding time for the patient arrives, the care team members and families are reminded to join. A phone call is automatically started if families reply requesting a phone call.

The purpose of this study was to understand and compare clinicians’ (including physicians, nurses, and advanced practice providers) perspectives and experiences (such as the impact on rounding efficiency, clinician satisfaction, trainee teaching, as well as facilitators and barriers in its implementation) about rounding before and after implementing Q-rounds to better facilitate multidisciplinary FCR. We hypothesized that providing real-time notification of care team members and families about the expected rounding times would lead to improved experiences and efficiency, without affecting the availability of teaching time for learners.

## Methods

This was a single-center survey study. We used a research design using quantitative analysis of survey results, supported by qualitative analysis of open-ended survey questions.

### Ethical Considerations

This study was reviewed and approved by the University of Minnesota Institutional Review Board (IRB-STUDY 00017619), and the IRB determined that this study involves no greater than minimal risk. Researchers who developed the software (MBP and JS) had conflict of interest policies managed by the University and were not allowed to access raw data.

### Settings

The study took place in the M Health Fairview Masonic Children’s Hospital 68-bed level IV NICU. Surveys were distributed in two 6-week phases: preimplementation (February 6, 2023-March 20, 2023) and postimplementation (June 14, 2023-July 27, 2023).

Rounds occur each morning and involve the clinical teams (attending, fellow, and resident physicians or advanced practice providers) working from a conference room. In addition to the attending physician and residents or advanced practice providers, the teams typically include a pharmacist, a dietitian, and sometimes a neonatology fellow. Before Q-rounds implementation, 1 team member would find or call the nurse for a patient when they were ready to round, and the nurse would bring the family with them if they were present in the room. This summoning to the conference room rounding approach is used in this setting for privacy, as there are shared patient rooms, preventing discussions at the bedside.

### Study Design

We developed a Research Electronic Data Capture (REDCap; Vanderbilt University) survey to elicit perceptions of nurse and family member presence on rounds, rounding length of time and efficiency, clinician communication, and trainee teaching experience, as well as participants’ demographics and roles. We had a mix of question styles including closed-ended multiple-choice questions, 5-point Likert-style questions, and open-ended questions. Likert scale questions included anchors of agreement (1 “strongly disagree” to 5 “strongly agree”), satisfaction (1 “very dissatisfied” to 5 “very satisfied”), or temporal assessment (1 “almost never” to 5 “almost always”) depending on the prompt. The survey used branching logic to present relevant questions to physicians, advanced practice providers, and nurses, with several common questions being asked from both groups. The number of questions ranged between 15 to 25 depending on the branching logic used. Each question asked the respondent to reflect over the last week when providing an answer. In the postimplementation survey, there were additional open-ended questions asking about perceptions of the implementation of the Q-rounds software. We expected that it would take approximately 10 to 15 minutes to complete the survey.

### Participants

All nurses, advanced practice providers, attending physicians, and trainee physicians (resident and fellow physicians) who work in the NICU were eligible to participate in the study. Participation was voluntary and anonymous, with no link to their email or other identifying information. In the instructions for the survey, people were instructed to take it only once, that is, 1 survey per 2-week period *within each phase* of the study.

### Intervention or Exposure

The intervention being evaluated in this study was the implementation of the Q-rounds software described in detail in the background section above. When a patient was admitted to the NICU, the nurse asked the family if they wished to receive SMS text message updates about when to expect the medical team for rounds. If they indicated yes, the nurse would indicate so in a flowsheet in the EHR, which would enroll the family in Q-rounds and they would subsequently receive notifications about rounding time and the ability to RSVP to join in person or remotely. Regardless of whether or not the family opted in to enroll, nurses would still receive notifications of rounding time. The electronic survey was created using REDCap tools hosted at the University of Minnesota [15,16].

### Data Collection

The surveys were sent to 180 potential participants on a rolling basis via email. For the trainee physicians, attending physicians, and advanced practice providers, they received 1 email if they had been on service in the prior 2 weeks, and 1 reminder email. For the nurses, the complexity and inconsistency of their schedules required sending the survey to the listserv of all nurses who work in the NICU and limiting responses via branching logic in the survey to only those who had worked in the study period. All nurses received 1 email each week during the study. All participants were asked to complete 1 survey per 2-week period in each study phase. All data were securely stored within Box [17] and REDCap [15,16].

### Data Analysis

#### Quantitative Analysis

Descriptive statistics (counts and percentages) were used to summarize survey questions for the preimplementation and postimplementation periods. Wilcoxon rank sum tests were used to compare the question responses between the periods. *P* values <.05 were considered statistically significant. SAS (version 9.4; SAS Institute) was used for the analysis.

#### Qualitative Analysis

The open-ended survey responses were analyzed using the rapid template analysis approach [18,19]. Template analysis is a structured approach in which researchers identify major domains covered in a data collection and provide summaries of participant responses for each domain. They then place each summary in a matrix to identify recurring themes under each domain. Coding was done by 2 coauthors (RFR and ESJ). The whole process was iterative until coders came to an agreement. We analyzed 59 and 60 open-ended survey responses, respectively, to each question, “What was most useful about using Q-rounds?” and “What would you suggest changing about Q-rounds to make it more useful?”

## Results

Q-rounds was successfully implemented during the crossover period of the study to notify nurses and families of when they were expected for rounds and to allow families to join remotely. The software was used for rounds daily during the intervention period.

A summary of survey respondents by role is provided in Table 1. There were 118 respondents in the preimplementation phase and 110 in the postimplementation phase. Nurses comprised most respondents in both phases: (n=66, 55.9% preimplementation and n=71, 64.5% postimplementation).

**Table 1.** Summary of clinical role by survey period.

	Preimplementation (n=118), n (%)	Postimplementation (n=110), n (%)
Attending physician	15 (12.7)	6 (5.5)
Advanced practice provider	24 (20.3)	24 (21.8)
Resident physician	9 (7.6)	5 (4.5)
Fellow physician	4 (3.4)	2 (1.8)
Nurse	66 (55.9)	71 (64.5)
Other	0 (0)	2 (1.8)

The analysis of the quantitative survey data is summarized in Table S1 in Multimedia Appendix 1. The additional questions in our survey, although results not discussed here, are included in Table S2 in Multimedia Appendix 1 for review.

The quantitative findings were supported by our qualitative analysis of the open-ended survey results of physicians, advanced practice providers, and nurses. Of the 110 participants of the postimplementation survey, 66 responded to at least one of the 2 open-ended questions that were thematically analyzed.

The most common coded response regarding usefulness of the software was for improved planning for rounds (n=20, 55%), followed by improved efficiency (n=6, 17%), parent attendance (n=6, 17%), and finally, nurse attendance (n=4, 11%). The most common coded response for suggestions for improvement was for improving the notification system (n=11, 37%), adding additional team members to be notified for rounds (n=10, 33%), improving the timing accuracy of the notifications (n=6, 20%), and lastly, improving education for team members and families regarding Q-rounds (n=3, 10%).

### Improved Perceived Attendance by Key Personnel

In the postimplementation period, there was an increase in the perception of nurse presence for the entirety of rounds, with the perception of the nurse being present “most” or “all the time” increasing from 48/105 (46%) to 24/36 (75%; *P*<.001). Similarly, there was an increase in the perception of the family being present for rounds, with clinicians indicating that family was present “at least sometimes” increasing from 45/106 (42%) to 36/36 (97%; *P*<.001). There was no significant difference in time spent waiting for nurses to arrive for FCR (*P*=.47) or the ability to provide daily family updates (*P*=.55). There was no difference in the perception of time waiting for nurses.

The above findings were further supported by the following quotes from the clinicians about their perception of significantly improved attendance:

*More opportunity for me to be part of family updates, whether listening in and showing/stating agreement with the resident, providing coaching for the resident during family update, or being able to direct/deliver the update.*

Attending physician 115

*Simply clicking a button to get the next patient’s nurse and family was so much easier than making calls.*

Attending physician 2

*Increased availability for RNs and Parents to join rounds; Improved expectations regarding rounds (i.e.: RN can see an estimated time for rounds and knows when to expect rounds to happen).*

Nurse practitioner 38

*Facilitating nurse communication/participation in a timely way.*

Attending physician 23

While there was an increased perceived opportunity for parents to join, survey participants cited the need for increased family education regarding the software and improvement in the timing of notifications for nurses and families:

*It was difficult to help families navigate how to add additional parent to Q-rounds.*

Attending MD 2

*[We need] better education to parents on how it works to encourage more involvement.*

Nurse practitioner 11

*[Would like] allowing for updated time calls when they decide to push rounds back or another patient rounds take really low*.

Nurse 88

*[Would be useful to have] calls for updates in expected time changes for rounds if more than 10 minutes different than originally anticipated*.

Nurse 57

A highly suggested improvement was also to add notifications for other team members:

*Notifying respiratory therapy would also be useful.*

Fellow MD 1

*Include other allied health staff.*

Attending MD 45

### Perceived Enhanced Rounding Efficiency and Planning

There was no change in perceived rounding length (*P*=.40), or the time spent discussing each patient (*P*=.54), which was also supported by the previous observational study [14]. Despite these findings, after implementing Q-rounds, there was an increase in clinicians’ perception of rounding efficiency with the percentage of clinicians who agreed with the statement that rounding was efficient, increasing from 56/105 (53%) to 62/84 (75%; *P*=.006). Similarly, more clinicians indicated they were satisfied with rounds after implementation of Q-rounds (62/84, 74%) compared to before implementation (59/105, 56%; *P*=.003). Importantly, there was no significant change in satisfaction with trainee teaching time (*P*=.82).

This was supported by a qualitative analysis theme identified highlighting the improved efficiency of the rounding process after the intervention:

*This decreases the amount of time spent updating families after rounds. It is a huge benefit in regard to workflow and time management.*

Nurse practitioner 71

*Q-rounds coordinates and calls the nurses and families for us leaving me more time to open charts and notes and prepare. This helps minimize time between patients.*

Nurse practitioner 44

Furthermore, there was improved perceived ability to have a plan for rounds, including having a rounding order and rough timeline of rounds. Examples of quotes supporting this theme include the following:

*Knowing a general timeline helped me not be in the thick of a time-consuming treatment/bath/educational moment so I could attend rounds.*

Nurse 86

*Organization of the rounding structure and personnel, keeping the team on focus.*

Nurse practitioner 36

## Discussion

### Principal Findings

This study surveyed clinicians and nurses on their perceptions of rounding practices before and after the implementation of novel rounding software. We found that the respondents had improved perceptions of rounding efficiency and satisfaction with rounding. We also found a perceived improvement in nurse and parent attendance. While there were suggestions for improving the software, they were mainly related to providing education, fine-tuning the notification timing, and including additional team members in the notifications. Both our quantitative analysis and coding of open-ended survey responses supported findings from our previous observational study, which showed that Q-rounds improved nurse and parent attendance [13]. Direct quotes from the clinicians further endorsed the value Q-rounds adds in terms of improving perceived efficiency of rounding practices and overall patient care delivery.

This approach directly addresses barriers to FCRs, such as giving nurses advanced notice of rounding timing and accommodating family schedules, not only by giving advance notice but also by allowing them to join via telephone [8,9]. Our results align with previous studies, showing that schedule-based FCR increases clinician satisfaction [12], and novel nursing contact strategies increase nurse attendance [20]. As with others, we found that families are highly interested in knowing when to expect the medical team for rounds [21], and explicit invitations help to increase caregiver participation [22]. Our intervention is novel, however, in that previous interventions typically focused on approaches at either getting more families or nurses to rounds, but not both [23]. Thus, Q-rounds was broadly regarded as an innovative mechanism for implementing FCR within the NICU, reinforcing prior evidence that FCR enhances the engagement and participation of patients and their families [7]. Our approach is novel in its ability to be seamlessly integrated into the EHR, directly contact parents via technology such as phone app updates, and allow for either in-person or phone family participation. By meeting families where they are, we hope to improve care participation across all socioeconomic backgrounds. We are planning future studies to look more deeply at demographic and social determinants of health factors that may point to this approach, allowing families to participate in FCR who are often unable to attend in the hospital. Future studies will also assess other potential benefits of the increase in multidisciplinary FCR afforded by this approach, including impacts on length of stay and patient satisfaction.

Beyond increasing the presence of key personnel during rounding and making rounds more effective and efficient, the primary goal of FCR is, as the name suggests, to engage families and ensure their presence in the decision-making process. Prior research has demonstrated that when families are more present during rounds and actively involved in shared decision-making, their satisfaction with both the care provided and with the physicians increases [7,8]. Although the referenced study was conducted in an urban midsized city setting, we can see a strong role for virtual or “Q-rounds” in rural environments as well as larger, high-stress, fast-paced cities with commuting delays. Especially in rural settings, various sociodemographic factors such as competing family responsibilities, time limitations, and transportation challenges can make in-person participation difficult. Virtual rounds allow patients and families to engage remotely, ensuring they remain involved in care despite these constraints. Additionally, virtual rounds are valuable in situations where exposure to patients must be minimized, for example, during pandemics, outbreaks of contagious conditions, or when caring for immunocompromised patients. Using a virtual rounding platform reduces infection risk while still maintaining a family-centered approach, regardless of whether the family can be physically present at the bedside.

In a follow-up observational study conducted 2 years after initial implementation, we evaluated whether the early benefits associated with the virtual rounding queue software were sustained over time [24]. We also examined its performance across 2 distinct rounding models: table rounds and walking rounds [24]. When comparing family attendance across the 2 rounding cohorts, overall family presence was similar; however, the walk rounding cohort showed a significantly higher rate of in-person attendance and a greater proportion of nurses present for the entirety of rounds [24]. Rounding duration did not significantly change across any of the table rounding cohorts over time, helping address persistent concerns that FCR reduces efficiency [24]. An important consideration for broader implementation is the feasibility of updating the virtual queue during walk rounds, where the team is physically moving from 1 bedside to the next. Centers that rely heavily on walk-around workflows may require modest adaptations, such as designating a specific team member to update the queue, using hands-free or voice-activated features, or some shortcut functionalities.

There are limitations to this study. This was an initial implementation, limited to rounding teams of the same unit in a single medical center. Future studies will aim to expand to additional units, teams, and medical centers to more fully understand the effect of Q-rounds. While our results found no significant difference in satisfaction regarding time to teach trainees, findings are constrained by the low number of resident physicians who completed the surveys. Further studies focusing on trainees, including medical students, residents, and fellows, are needed to better understand their educational benefits from Q-rounds. Our study did not incorporate family feedback, which will be obtained in a future study. The fact that we were unable to individually target nurses to participate and had to use a large listserv of the entire NICU staff, including those who only work night shifts, prohibited us from being able to calculate a true response rate. We also had a lower response rate (66/110, 60%) for clinicians who answered the open-ended qualitative questions as compared to the quantitative survey questions, which may have limited our thematic analysis. It is worth noting that the clinicians asked to continue to use this approach for rounding in the NICU after the study was complete, and it has since become the standard for rounding across all 4 teams in the 68-bed NICU as well as other units in the same hospital.

At the time of this study, Q-rounds was an internally developed, grant-supported research tool implemented within a single health system, and there were no direct institutional costs associated with its use. Since completion of this pilot, the technology has undergone commercialization and is now available for use at other institutions. Generalizability may therefore be influenced by local resource considerations. For example, at the time of this study, Q-rounds was only able to integrate with Epic’s EHR. Institutions seeking to replicate this approach would need to either develop a comparable internal tool or adopt an existing solution, both of which carry financial and operational implications.

### Conclusions

Q-rounds provides an innovative approach that addresses multiple previously identified hurdles to FCR, including real-time updated scheduling and the ability to work with families’ schedules by creating an option that allows families to confirm attendance by phone or in person. Clinicians perceived having a technical solution that provides real-time updates to nurses and families about the timing of rounds, with the ability for families to join remotely or in person, as an effective way to facilitate multidisciplinary FCR. While previously shown to improve nurses’ participation, here we showed that Q-rounds was associated with improved clinician perceptions of efficiency, increased nurse and family participation in rounds, and increased satisfaction with rounds. Future evaluation of Q-rounds integrated into other EHRs and the development of additional features to support multidisciplinary FCR, including virtual family participation by video, enhanced interpreter coordination, and options for families to submit questions in advance of rounds represent potential directions for broader implementation.

## Supplementary material

10.2196/78547 Multimedia Appendix 1 Summary of pre- and postsurvey clinician responses and additional survey questions.

## Abbreviations

EHR: electronic health record
FCR: family-centered round
NICU: neonatal intensive care unit
REDCap: Research Electronic Data Capture
