# Supplementary material for: Impact of a Real-Time Virtual Rounding Queue on Neonatal Intensive Care Unit Rounding Practices: Survey Study of Clinicians’ Perceptions
Source: JMIR Pediatr Parent. 2026 Jun 18;9:e78547. doi: 10.2196/78547 (PMC13278618; doi:10.2196/78547)
Supplement: Multimedia Appendix 1 [file pediatrics-v9-e78547-s001.docx]

Supplemental Table 1: Summary of Pre- and Post-Clinician Survey Responses

| Question | Answer Choices | Pre-Implementation Results | Post-Implementation Results | P-value † |
| --- | --- | --- | --- | --- |
| What is the average amount of time you spend per day discussing each patient on the NICU rounds? | *n (%)*  Less than 10 minutes  10 to 20 minutes  21 to 30 minutes  More than 30 minutes | n=115  29 (25.2)  76 (66.1)  9 (7.8)  1 (0.9) | n=110  25 (22.7)  73 (66.4)  11 (10.0)  1 (0.9) | 0.544 |
| Thinking back on your last week of clinical service, how long did NICU rounds take on average? | *n (%)*  1 to 2 hours  2 to 3 hours  3 to 4 hours  More than 4 hours | n=50  2 (4.0)  19 (38.0)  26 (52.0)  3 (6.0) | n=39  0  16 (41.0)  16 (41.0)  7 (17.9) | 0.405 |
| How often was the nurse there for the entire rounding discussion on their patient (i.e., from start to finish)? | n (%)  1=Almost never  2=Rarely  3=Sometimes  4=Most of the time  5=Almost all of the time    Mean (SD)  Median (IQR) | n=105  5 (4.8)  17 (16.2)  35 (33.3)  42 (40.0)  6 (5.7)    3.3 (1.0)  3.0 (3.0-4.0) | n=36  0  1 (2.8)  8 (22.2)  22 (61.1)  5 (13.9)    3.9 (0.7)  4.0 (3.5-4.0) | **<0.001** |
| How often did the team have to wait for the nurse to arrive when it was time to discuss their patient on rounds? | n (%)  1=Almost never  2=Rarely  3=Sometimes  4=Most of the time  5=Almost all of the time    Mean (SD)  Median (IQR) | n=46  4 (8.7)  3 (6.5)  22 (47.8)  12 (26.1)  5 (10.9)    3.2 (1.0)  3.0 (3.0-4.0) | n=36  0  7 (19.4)  19 (52.8)  7 (19.4)  3 (8.3)    3.2 (0.8)  3.0 (3.0-4.0) | 0.471 |
| How often did the team have to round on a patient without the nurse being able to be present at all? | n (%)  1=Almost never  2=Rarely  3=Sometimes  4=Most of the time  5=Almost all of the time    Mean (SD)  Median (IQR) | n=46  2 (4.3)  14 (30.4)  30 (65.2)  0  0    2.6 (0.6)  3.0 (2.0-3.0) | n=36  8 (22.2)  16 (44.4)  11 (30.6)  1 (2.8)  0    2.1 (0.8)  2.0 (2.0-3.0) | **0.003** |
| How often was a member of the patient’s family present for rounds on each patient? | n (%)  1=Almost never  2=Rarely  3=Sometimes  4=Most of the time  5=Almost all of the time    Mean (SD)  Median (IQR) | n=106  21 (19.8)  40 (37.7)  43 (40.6)  1 (0.9)  1 (0.9)    2.3 (0.8)  2.0 (2.0-3.0) | n=36  0  1 (2.8)  30 (83.3)  4 (11.1)  1 (2.8)    3.1 (0.5)  3.0 (3.0-3.0) | **<.0001** |
| How often were you personally able to provide a daily update to your patient’s family? | n (%)  1=Almost never  2=Rarely  3=Sometimes  4=Most of the time  5=Almost all of the time    Mean (SD)  Median (IQR) | n=105  3 (2.9)  8 (7.6)  28 (26.7)  33 (31.4)  33 (31.4)    3.8 (1.1)  4.0 (3.0-5.0) | n=36  0  1 (2.8)  12 (33.3)  10 (27.8)  13 (36.1)    4.0 (0.9)  4.0 (3.0-5.0) | 0.546 |
| Thinking back on your last week of clinical service, how much do you agree or disagree with the following statement: Rounding in the NICU was efficient. | n (%)  1=Strongly disagree  2=Disagree  3=Neither disagree or agree  4=Agree  5=Strongly agree    Mean (SD)  Median (IQR) | n=105  4 (3.8)  18 (17.1)  27 (25.7)  49 (46.7)  7 (6.7)    3.4 (1.0)  4.0 (3.0-4.0) | n=84  7 (8.3)  2 (2.4)  12 (14.3)  54 (64.3)  9 (10.7)    3.7 (1.0)  4.0 (3.5-4.0) | **0.006** |
| Thinking back on your last week of service, how much do you agree or disagree with the following statement: I was happy with the amount of time spent teaching during rounds. | n (%)  1=Strongly disagree  2=Disagree  3=Neither disagree or agree  4=Agree  5=Strongly agree    Mean (SD)  Median (IQR) | n=46  1 (2.2)  12 (26.1)  11 (23.9)  19 (41.3)  3 (6.5)    3.2 (1.0)  3.0 (2.0-4.0) | n=36  1 (2.8)  6 (16.7)  13 (36.1)  13 (36.1)  3 (8.3)    3.3 (1.0)  3.0 (3.0-4.0) | 0.817 |
| What is your level of satisfaction with how rounding works in NICU settings when it comes to the presence of essential team members including the patient’s family? | n (%)  1=Very dissatisfied  2=Dissatisfied  3=Neither dissatisfied or satisfied  4=Satisfied  5=Very satisfied    Mean (SD)  Median (IQR) | n=105  0  20 (19.0)  26 (24.8)  50 (47.6)  9 (8.6)    3.5 (0.9)  4.0 (3.0-4.0) | n=84  0  3 (3.6)  19 (22.6)  50 (59.5)  12 (14.3)    3.8 (0.7)  4.0 (3.0-4.0) | **0.003** |

† P-value is from a Wilcoxon rank sum test. P < 0.05 considered statistically significant.
 Note: The sample sizes differed by question as certain questions due to the use of branching logic within the survey, where certain questions were only presented to a subset of participants based on prior responses. Additionally, not all participants completed every question, resulting in some missing data.

Supplemental Table 2: Additional Survey Questions

| Question | Answer choices | Answers n (%) |
| --- | --- | --- |
| Thinking back on your last week of clinical service, how many days did your team use Q-rounds to coordinate rounding with at least one patient? | 0 day  1 day  2 days  3 days  4 days  5 days  6 days  7 days | 3 (3.6)  9 (11)  13 (15,5)  **24 (28.6)**  6 (7.1%)  2 (2.4%)  3 (3.6)  **24 (28.6%)** |
| I found Q-rounds to be a useful tool. | 1=Strongly disagree  2=Disagree  3=Neutral  4=Agree  5=Strongly agree | 1 (1.2)  0 (0)  4 (4.8)  **53 (63)**  23 (27.4) |
| I thought Q-rounds were easy to use. | 1=Strongly disagree  2=Disagree  3=Neutral  4=Agree  5=Strongly agree | 0 (0)  0 (0)  7 (8.3)  **56 (66.6)**  18 (21.4) |
| Do you want the NICU to continue to use Q-rounds in the future? | Yes, I would want the NICU to use Q-rounds in the future, as-is, without any further enhancements 2  Yes, I would want the NICU to use Q-rounds in the future after upgrading it with some enhancements  I am ambivalent about using Q-rounds in the NICU  No, I would not want the NICU to use Q-rounds | 27 (32.1)  **52 (70)**  4 (4.8)  1 (1.2) |
